# Supplementary material for: Pyruvate Dehydrogenase Kinase 1 inhibition mediated oxidative phosphorylation enhancement in cartilage promotes osteoarthritis progression
Source: BMC Musculoskelet Disord. 2023 Jul 20;24:597. doi: 10.1186/s12891-023-06585-6 (PMC10357736; doi:10.1186/s12891-023-06585-6)
Supplement: Supplementary file 1 — Additional file 1. [file 12891_2023_6585_MOESM1_ESM.zip › BP.Gsea.1653623667859/GOBP_PURINE_RIBONUCLEOSIDE_METABOLIC_PROCESS.html]

Details for gene set GOBP\_PURINE\_RIBONUCLEOSIDE\_METABOLIC\_PROCESS[GSEA]

|  || Dataset | 55457\_collapsed\_to\_symbols.cls.cls #OA\_versus\_normal.cls.cls #OA\_versus\_normal\_repos |
| Phenotype | cls.cls#OA\_versus\_normal\_repos |
| Upregulated in class | OA |
| GeneSet | GOBP\_PURINE\_RIBONUCLEOSIDE\_METABOLIC\_PROCESS |
| Enrichment Score (ES) | 0.5135709 |
| Normalized Enrichment Score (NES) | 1.5814457 |
| Nominal p-value | 0.019002376 |
| FDR q-value | 0.28189036 |
| FWER p-Value | 1.0 |
Table: GSEA Results Summary

  

Fig 1: Enrichment plot: GOBP\_PURINE\_RIBONUCLEOSIDE\_METABOLIC\_PROCESS      
 Profile of the Running ES Score & Positions of GeneSet Members on the Rank Ordered List

  

| SYMBOL | TITLE | RANK IN GENE LIST | RANK METRIC SCORE | RUNNING ES | CORE ENRICHMENT || 1 | DGUOK | deoxyguanosine kinase [Source:HGNC Symbol;Acc:HGNC:2858] | 96 | 0.917 | 0.1425 | Yes |
| 2 | ADK | adenosine kinase [Source:HGNC Symbol;Acc:HGNC:257] | 127 | 0.878 | 0.2838 | Yes |
| 3 | ENPP4 | ectonucleotide pyrophosphatase/phosphodiesterase 4 [Source:HGNC Symbol;Acc:HGNC:3359] | 822 | 0.563 | 0.3220 | Yes |
| 4 | ICMT | isoprenylcysteine carboxyl methyltransferase [Source:HGNC Symbol;Acc:HGNC:5350] | 1140 | 0.488 | 0.3772 | Yes |
| 5 | APRT | adenine phosphoribosyltransferase [Source:HGNC Symbol;Acc:HGNC:626] | 1178 | 0.481 | 0.4529 | Yes |
| 6 | PTGDR | prostaglandin D2 receptor [Source:HGNC Symbol;Acc:HGNC:9591] | 1904 | 0.372 | 0.4575 | Yes |
| 7 | ADA2 | adenosine deaminase 2 [Source:HGNC Symbol;Acc:HGNC:1839] | 1955 | 0.367 | 0.5136 | Yes |
| 8 | MTAP | methylthioadenosine phosphorylase [Source:HGNC Symbol;Acc:HGNC:7413] | 3324 | 0.219 | 0.4432 | No |
| 9 | NT5E | 5'-nucleotidase ecto [Source:HGNC Symbol;Acc:HGNC:8021] | 3515 | 0.201 | 0.4613 | No |
| 10 | ACP3 | acid phosphatase 3 [Source:HGNC Symbol;Acc:HGNC:125] | 6271 | -0.025 | 0.2516 | No |
| 11 | HPRT1 | hypoxanthine phosphoribosyltransferase 1 [Source:HGNC Symbol;Acc:HGNC:5157] | 6954 | -0.076 | 0.2111 | No |
| 12 | ADA | adenosine deaminase [Source:HGNC Symbol;Acc:HGNC:186] | 8105 | -0.163 | 0.1486 | No |
| 13 | XDH | xanthine dehydrogenase [Source:HGNC Symbol;Acc:HGNC:12805] | 8366 | -0.183 | 0.1583 | No |
| 14 | GNMT | glycine N-methyltransferase [Source:HGNC Symbol;Acc:HGNC:4415] | 9208 | -0.248 | 0.1337 | No |
| 15 | NT5C2 | "5'-nucleotidase, cytosolic II [Source:HGNC Symbol;Acc:HGNC:8022]" | 9595 | -0.283 | 0.1500 | No |
| 16 | PNP | purine nucleoside phosphorylase [Source:HGNC Symbol;Acc:HGNC:7892] | 9657 | -0.288 | 0.1923 | No |
| 17 | AMD1 | adenosylmethionine decarboxylase 1 [Source:HGNC Symbol;Acc:HGNC:457] | 10415 | -0.363 | 0.1929 | No |
Table: GSEA details [plain text format]

  

Fig 2: GOBP\_PURINE\_RIBONUCLEOSIDE\_METABOLIC\_PROCESS      
 Blue-Pink O' Gram in the Space of the Analyzed GeneSet

  

Fig 3: GOBP\_PURINE\_RIBONUCLEOSIDE\_METABOLIC\_PROCESS: Random ES distribution      
 Gene set null distribution of ES for **GOBP\_PURINE\_RIBONUCLEOSIDE\_METABOLIC\_PROCESS**
